# Safety of recombinant thrombomodulin during percutaneous transhepatic gallbladder drainage in patients with biliary tract infections and disseminated intravascular coagulation: A retrospective cohort study

**DOI:** 10.1097/MD.0000000000047869

**Published:** 2026-02-28

**Authors:** Tetsushi Azami, Yuichi Takano, Naoki Tamai, Jun Noda, Fumitaka Niiya, Masatsugu Nagahama

**Affiliations:** a Department of Medicine, Division of Gastroenterology, Showa Medical University Fujigaoka Hospital, Yokohama, Kanagawa, Japan.

**Keywords:** biliary tract infections, disseminated intravascular coagulation, percutaneous transhepatic gallbladder drainage, recombinant thrombomodulin

## Abstract

Biliary tract infections complicated by disseminated intravascular coagulation (DIC) require urgent source control and careful correction of coagulopathy. Recombinant thrombomodulin (rTM) is widely used for DIC in Japan, but its perioperative safety in patients undergoing percutaneous transhepatic gallbladder drainage (PTGBD) has not been fully clarified. This study aims to determine whether perioperative rTM administration increases the risk of PTGBD-related adverse events (AEs) in patients with biliary tract infections and DIC. This retrospective cohort study included patients with biliary tract infections and DIC who underwent PTGBD between January 2017 and August 2025 at a single tertiary center. Patients who died within 24 hours or who initiated rTM more than 3 days after PTGBD were excluded. The primary outcome was PTGBD-related AEs. Secondary outcomes included bleeding-related AEs, 28-day mortality, DIC resolution on Day 7, and intensive care unit length of stay. Bayesian sensitivity analyses and stratified analyses based on concomitant therapies were also performed. A total of 30 patients were included (19 received rTM and 11 did not). PTGBD-related AEs occurred in 21.1% of the rTM group and 18.2% of the non-rTM group. No bleeding-related AEs were observed in either group. Bayesian analysis estimated upper 95% credible limits of 2.8% and 3.1% for PTGBD-related bleeding in the rTM and non-rTM groups, respectively. Secondary outcomes – including 28-day mortality, DIC resolution on Day 7, and intensive care unit stay – showed no meaningful differences between groups, and stratified analyses yielded consistent results. Perioperative rTM use during PTGBD was not associated with an increased incidence of PTGBD-related AEs, including bleeding. These findings indicate that rTM may be administered without substantial additional procedural risk in patients with biliary tract infections and DIC, although larger studies are needed to validate its safety.

## 1. Introduction

Source control via biliary drainage is essential for treating biliary tract infections. In severe cases, disseminated intravascular coagulation (DIC) may occur. Because such patients are considered to be at high risk of bleeding, endoscopic transpapillary drainage without endoscopic sphincterotomy is the first-line method of biliary drainage.^[1]^ However, in severe cases, endoscopic treatment is often difficult owing to the patient’s condition, and percutaneous drainage methods such as percutaneous transhepatic gallbladder drainage (PTGBD) or percutaneous transhepatic biliary drainage (PTBD) may be considered.

In biliary tract infections complicated by DIC, in addition to urgent drainage, management of coagulopathy is key to treatment. Recombinant human soluble thrombomodulin (rTM) has been reported to be useful in the treatment of sepsis-associated DIC, with evidence suggesting improvements in DIC parameters and prognosis.^[2,3]^ rTM promotes thrombin-mediated activation of protein C. Activated protein C, with protein S as a cofactor, inactivates procoagulant factors Va and VIIIa, thereby suppressing thrombin generation and inhibiting the coagulation cascade. Owing to this mechanism of action, rTM administration is considered to increase bleeding risk.^[4]^

Reports on the utility of rTM in biliary tract infections also exist.^[5–8]^ However, in situations where bleeding risk is a major concern, such as PTGBD, rTM administration is sometimes avoided. Nevertheless, a randomized controlled trial and meta-analysis have suggested that rTM administration does not clearly increase the risk of bleeding.^[2,9]^ In addition, recent retrospective studies in patients with sepsis-induced DIC due to gastroenterological diseases have suggested that the incidence of serious bleeding adverse events (AEs) during rTM administration is low, even among cases requiring invasive procedures such as surgery or percutaneous drainage.^[10,11]^ However, although rTM may be safely administered during the perioperative period of PTGBD, it has not yet been validated.

The aim of this retrospective study was to investigate the impact of rTM on the safety of PTGBD in the perioperative setting.

## 2. Methods

### 2.1. Study design and participants

This single-center retrospective study was conducted using data from medical records. Participants who underwent PTGBD for biliary tract infections at Showa Medical University Fujigaoka Hospital between January 1, 2017, and August 31, 2025, were eligible. The inclusion criterion was the presence of DIC. The exclusion criteria were death within 24 hours and initiation of rTM administration more than 3 days after PTGBD. Registered participants were divided into rTM (with rTM administration) and non-rTM (without administration) groups for comparison.

This study was conducted in accordance with the Declaration of Helsinki and was approved by the Showa Medical University Research Ethics Review Board (approval number 2025-0351; registration date October 31, 2025).

### 2.2. Outcomes and definitions

The primary outcome was PTGBD-related AEs. Secondary outcomes were PTGBD-related bleeding, 28-day mortality, DIC resolution on Day 7, and length of intensive care unit (ICU) stay.

AEs were evaluated using the Clavien–Dindo classification.^[12]^ DIC was diagnosed based on the Japanese Association for Acute Medicine DIC criteria,^[13]^ and biliary tract infections and organ dysfunction were evaluated according to the Tokyo Guidelines 2018.^[1]^ For participants who were not admitted to the ICU, length of stay was recorded as 0 days.

### 2.3. Administration of rTM and details of the PTGBD procedure

The decision to administer rTM, as well as its timing and dosage, was made at the discretion of the attending physician.

Prior to puncture, 15 mg of pentazocine was administered intravenously as an analgesic, and local anesthesia with lidocaine was administered around the puncture site. Doppler ultrasound was used to confirm that the absence of intervening blood vessels was present before the puncture. Subsequently, an 18G needle (Hanaco Medical, Saitama, Japan) was inserted into the gallbladder under ultrasound guidance, and a small amount of bile was aspirated. A 0.035-inch guidewire (Radifocus, Terumo, Tokyo, Japan) was then placed in the gallbladder under fluoroscopy. After a small skin incision and dilation using a dilator, either a 6-Fr or 8-Fr pigtail catheter (UreSil L.C.C., Skokie) was inserted.

### 2.4. Statistical analysis

Continuous variables were expressed as medians and interquartile ranges, and categorical variables as frequencies (%). The standardized mean difference (SMD) was presented as a reference index for comparing baseline characteristics between groups.

For binary outcomes, the risk difference and relative risk (RR) with 95% confidence intervals (CIs) were calculated, and Fisher exact test was applied. In cases with few events and the possibility of complete separation, Firth logistic regression was used.

For bleeding-related AEs, 95% CIs were calculated using the Clopper–Pearson method, and sensitivity analysis was performed using Bayesian estimation. Based on previous reports, the PTGBD-related bleeding rate was assumed to be 1%.^[14]^ For the prior distribution, a beta (0.5, 49.5) distribution was set, assuming a mean of 1% and an effective sample size of 50. The posterior distribution, obtained by combining the prior distribution with the observed data, was used to calculate the upper 95% CI.

For continuous outcomes, the Mann–Whitney *U* test was used, and the Hodges–Lehmann method was applied to calculate the median difference between groups and its 95% CI.

As a sensitivity analysis, stratified analyses were conducted according to whether antithrombin III preparations or heparin were administered perioperatively. For binary outcomes, incidence rates were calculated for each stratum, and the common RR and 95% CI were estimated using the Mantel–Haenszel method. When the number of events was extremely small, only the risk difference and 95% CI were presented. For continuous outcomes, stratified medians (interquartile ranges) were calculated, and the van Elteren test was used to assess overall intergroup differences. Missing data were evaluated for all variables. Because the proportion of missing values was very small (<5%) and the pattern was deemed completely at random, a complete-case analysis was adopted without the use of imputation. A 2-sided *P*-value <.05 was considered statistically significant. Data analyses were performed using R version 4.0.3 (R Foundation for Statistical Computing, Vienna, Austria).

## 3. Results

### 3.1. Clinical characteristics of the participants

**Figure 1.**
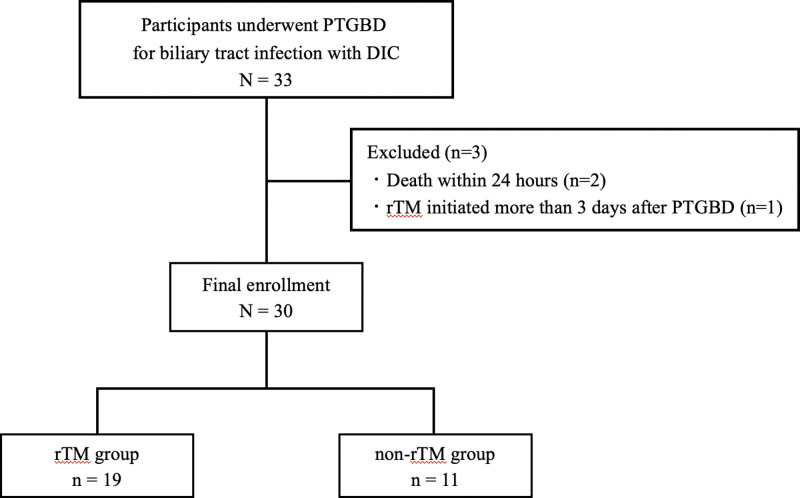
Flow chart of participant enrollment. The diagram depicts patient selection for the study cohort of those undergoing percutaneous transhepatic gallbladder drainage (PTGBD) for biliary tract infection with disseminated intravascular coagulation. Three patients were excluded: 2 due to death within 24 hours and 1 due to delayed initiation of recombinant thrombomodulin (rTM). DIC = disseminated intravascular coagulation, PTGBD = percutaneous transhepatic gallbladder drainage, rTM = recombinant thrombomodulin.

During the study period, PTGBD was performed in 33 patients with biliary tract infections complicated by DIC. Three cases were excluded: 2 deaths within 24 hours and 1 case in which rTM administration was initiated more than 3 days after PTGBD. Finally, 30 participants were enrolled, with 19 in the rTM group and 11 in the non-rTM group (Fig. 1). All cases were classified as severe.

The standard rTM dose was 380 U/kg; however, in 2 cases, it was reduced to 130 U/kg according to renal function. In 6 cases, rTM was started before PTGBD, and the median duration of administration was 4 [3–5] days.

Baseline characteristics are shown in Table 1. The median age was 78 (68–86) years in the rTM group and 85 (83–89) years in the non-rTM group, with the latter being significantly older (*P* = .045, SMD = -0.86). Platelet count was 7.5 (6.0–9.1) × 10^4^/μL in the rTM group and 9.0 (7.7–12.4) × 10^4^/μL in the non-rTM group, significantly higher in the non-rTM group (*P* = .013, SMD = -0.99).

**Table 1:** Baseline characteristics of the participants.

	rTM group (n = 19)	non-rTM group (n = 11)	*P*-value	SMD
Age (year), median (IQR)	78.0 (68.0–85.5)	85.0 (82.5–88.5)	.045	−0.86
Sex (male), n (%)	10 (52.6)	7 (63.6)	.708	−0.22
CCI, median (IQR)	7 (4–10)	7 (7–9)	.68	−0.21
ASA-PS, median (IQR)	3.0 (2.0–3.0)	3.0 (2.5–4.0)	.291	−0.43
Antithrombotic medication, n (%)	5 (26.3)	4 (36.4)	.687	−0.22
Other treatments for DIC				
Heparin, n (%)	2 (10.5)	2 (18.2)	.611	−0.22
Antithrombin Ⅲ, n (%)	5 (26.3)	1 (9.1)	.372	0.45
Chronic kidney disease, n (%)	4 (21.1)	1 (9.1)	.626	0.33
Hematological disease, n (%)	0	0	–	–
Chronic liver disease, n (%)	0	0	–	–
Etiology				
Cholecystitis, n (%)	9 (47.4)	5 (45.5)	1	0.04
Gallbladder stone, n	9	5		
Cholangitis, n (%)	10 (52.6)	6 (54.5)		
Common bile duct stone, n	9	4		
Pancreatic cancer, n	1	1		
Biliary stent obstruction, n	0	1		
Time to PTGBD (hour), median (IQR)	10.0 (5.0–18.0)	6.0 (4.5–15.0)	.62	0.28
PTGBD catheter diameter (8Fr), n (%)	4 (21.1)	1 (9.1)	.626	0.33
ICU admission, n (%)	14 (73.7)	6 (54.5)	.425	0.4
WBC (/μL), median (IQR)	16,060 (11,725–21,690)	16,860 (13,295–22,250)	.667	−0.10
Plt (x104/μL), median (IQR)	7.5 (6.0–9.1)	9.0 (7.7–12.4)	.013	−0.99
PT-INR, median (IQR)	1.4 (1.2–1.6)	1.3 (1.2–1.4)	.731	0.28
D-dimer (μg/mL), median (IQR)	14.6 (6.2–31.5)	16.7 (6.0–23.0)	.783	0.49
DIC score, median (IQR)	5 (4–7)	4 (4–5)	.099	0.69
SOFA score, median (IQR)	6 (5–8)	5 (4.5–7)	.382	0.34
Number of dysfunctional organs, median (IQR)	2 (1–3)	2 (1–2.5)	.557	0.32
Cardiovascular dysfunction, n (%)	12 (63.2)	3 (27.3)	.128	0.77
Neurological dysfunction, n (%)	5 (26.3)	3 (27.3)	1	0.022
Respiratory dysfunction, n (%)	5 (26.3)	3 (27.3)	1	0.02
Renal dysfunction, n (%)	7 (36.8)	4 (36.4)	1	0.01
Hepatic dysfunction, n (%)	6 (31.6)	2 (18.2)	.672	0.31
Hematological dysfunction, n (%)	15 (78.9)	6 (54.5)	.225	0.54

ASA-PS = American Society of Anesthesiologists physical status classification, CCI = Charlson comorbidity index, DIC = disseminated intravascular coagulation, ICU = intensive care unit, IQR = interquartile range, non-rTM group = participants who did not receive recombinant thrombomodulin, Plt = platelet count, PTGBD = percutaneous transhepatic gallbladder drainage, PT-INR = prothrombin time-international normalized ratio, rTM group = participants who received recombinant thrombomodulin, SMD = standardized mean difference, SOFA = sequential organ failure assessment, WBC = white blood cell count.

No significant differences were observed between the 2 groups in Charlson Comorbidity Index, ASA-PS, PT-INR, sex, use of antithrombotic agents, concomitant use of heparin or antithrombin III, chronic kidney disease, proportion of cholecystitis/cholangitis, PTGBD catheter diameter, or ICU admission rate. No participants with hematological or chronic liver disease were included in either group.

### 3.2. Outcomes

Outcomes are summarized in Table 2. For the primary outcome, PTGBD-related AEs occurred in 4 cases (21.1%) in the rTM group (two cases of bile leakage Grade I and 2 cases of dislodgement) and 2 cases (18.2%) in the non-rTM group (one case of shock Grade II and 1 case of wound infection Grade I). No significant difference was detected (RD + 2.9%, 95% CI −26.2 to 32.0; RR 1.16, 95% CI 0.25–5.41; *P* = 1.000).

**Table 2:** Comparison of outcomes between groups.

Outcomes	rTM group (n = 19)	non-rTM group (n = 11)	*P*-value
PTGBD-related adverse events, n (%)	4 (21.1)	2 (18.2)	1
PTGBD-related adverse bleeding events, n	0	0	–
DIC resolution on day 7, n (%)	16 (84.2)	10 (90.9)	1
28-day mortality, n (%)	2 (10.5)	2 (18.2)	.596
length of ICU stay (day), median (IQR)	4 (0–10)	0 (0–6)	.241

DIC = disseminated intravascular coagulation, ICU = intensive care unit, IQR = interquartile range, non-rTM group = participants who did not receive recombinant thrombomodulin, PTGBD = percutaneous transhepatic gallbladder drainage, rTM group = participants who received recombinant thrombomodulin.

No PTGBD-related bleeding occurred in either group, and logistic regression analysis could not be applied owing to complete separation of events. The 95% CI using the Clopper–Pearson method was 0% to 17.6% for the rTM group and 0% to 28.5% for the non-rTM group. Using Bayesian sensitivity analysis (prior Beta [0.5, 49.5], effective sample size = 50, mean 1%), the upper 95% CI limits were 2.8% for the rTM group and 3.1% for the non-rTM group.

Furthermore, 28-day mortality occurred in 2 cases (10.5%) in the rTM group and 2 cases (18.2%) in the non-rTM group, with no significant intergroup difference (RD −7.7%, 95% CI −35.6 to 20.1; RR 0.58, 95% CI 0.11–3.10; *P* = .596).

DIC resolution on Day 7 occurred in 16 cases (84.2%) in the rTM group and 10 cases (90.9%) in the non-rTM group, again without a meaningful difference (RD −6.7%, 95% CI −32.4 to 19.0; RR 0.93, 95% CI 0.74–1.18; *P* = 1.000).

The median (IQR) length of ICU stay was 4 (0–10) days in the rTM group and 0 (0–6) days in the non-rTM group. The Hodges–Lehmann median difference was + 2 days (95% CI −6 to 2; *P* = .241).

Stratified analysis was performed according to perioperative administration of antithrombin III preparations or heparin. For the primary outcome (PTGBD-related AEs), among those who did not receive concomitant therapy, 4/13 cases (30.8%) occurred in the rTM group and 2/8 cases (25.0%) in the non-rTM group, whereas no AEs occurred in those who received concomitant therapy. The pooled odds ratio obtained using the Mantel–Haenszel method was 1.33 (95% CI 0.18–9.72; *P* = .78).

Regarding 28-day mortality, among those who did not receive concomitant therapy, survival was achieved in 12/13 participants (92.3%) in the rTM group and 6/8 participants (75.0%) in the non-rTM group; among those who received concomitant therapy, survival was achieved in 5/6 participants (83.3%) in the rTM group and 3/3 participants (100%) in the non-rTM group. The pooled odds ratio was 0.54 (95% CI 0.07–4.41; *P* = .57).

For DIC resolution on Day 7, among those who did not receive concomitant therapy, 11/13 (84.6%) in the rTM group and 7/8 (87.5%) in the non-rTM group showed resolution; among those who received concomitant therapy, 5/6 participants (83.3%) in the rTM group and 3/3 participants (100%) in the non-rTM group showed resolution. The pooled odds ratio was 0.52 (95% CI 0.05–5.86; *P* = .61).

The median (IQR) length of ICU stay was 4 (0–10) days in the rTM group and 0 (0–5) days in the non-rTM group that did not receive concomitant therapy, and 6.5 (5.25–7.0) days in the rTM group and 6.0 (5.5–7.5) days in the non-rTM group that received concomitant therapy. No significant intergroup differences were observed in the pooled analysis using the van Elteren test (*P* >.2).

## 4. Discussion

In this study, no differences were observed in PTGBD-related AEs between the rTM and non-rTM groups during the perioperative period; notably, no bleeding-related AEs occurred in the rTM group. The 95% CIs derived using the Clopper–Pearson method were 0% to 17.6% and 0% to 28.5% in the rTM and non-rTM groups, respectively. Furthermore, when the PTGBD-related bleeding rate was set at 1% based on previous reports, the upper 95% CI limits were 2.8% and 3.1% in the rTM and non-rTM groups, respectively. Sensitivity analysis evaluating the influence of concomitant antithrombin III preparations or heparin indicated that such therapy was more commonly administered in the rTM group; however, the results remained consistent. These findings suggest that perioperative rTM administration during PTGBD does not increase bleeding-related AEs.

This observation is consistent with previous reports. Fujikawa et al reported, in a retrospective study of patients with sepsis-induced DIC that included a substantial proportion of cases requiring invasive procedures such as surgery or percutaneous drainage, that the incidence of bleeding AEs during rTM administration was relatively low (5.8%), and that serious bleeding AEs were rare.^[10]^ Moreover, Nagata et al reported that no severe perioperative bleeding AEs were observed among 32 patients with sepsis-induced DIC who received rTM following emergency abdominal surgery.^[11]^ Beyond safety considerations, although evidence remains limited, several reports have suggested the potential utility of rTM in treating biliary tract infections. Okuda et al retrospectively examined rTM use in cholangitis complicated by DIC among 173 participants in the rTM group and 111 in the non-RTM group. They reported significantly higher DIC resolution rate on Day 7 (89% vs 71%) and 28-day survival (94% vs 83%) in the rTM group.^[5]^ Ogura et al reported similar findings in patients with cholecystitis and DIC; in their retrospective study of 17 participants in the rTM group and 25 in the non-rTM group, the Day 7 DIC resolution rate was significantly higher in the rTM group (94.1% vs 68.0%).^[6]^

However, in the present study, both the main and sensitivity analyses showed no significant intergroup differences in 28-day mortality, DIC resolution on Day 7, or length of ICU stay. Platelet count at admission was significantly lower in the rTM group, and although not statistically significant, the sequential organ failure assessment score and cardiovascular dysfunction tended to be more frequent in the rTM group. Because rTM administration was determined by the attending physician, the rTM group may have included more severe cases than the non-rTM group. Therefore, the interpretation of 28-day mortality, DIC resolution on Day 7, or length of ICU stay should be considered only as reference points.

In this study, PTGBD was performed in patients with cholangitis, and no hemorrhagic AEs were observed. Endoscopic treatment is often difficult in patients with biliary infections complicated by DIC, and percutaneous drainage may therefore be considered as an alternative. Although PTBD is generally regarded as the first-line procedure for cholangitis, its technical success rate is 86% in patients with bile duct dilatation and 63% in those without.^[15]^ In patients with DIC, respiratory failure or impaired consciousness may preclude adequate cooperation, such as breath-holding, thereby increasing the technical difficulty. Moreover, PTBD has been reported to have a relatively high AE rate of 13% (34/253).^[16]^ When the level of obstruction is located distal to the cystic duct junction, the drainage efficacy of PTGBD for obstructive jaundice has been reported to be comparable to that of PTBD.^[17–21]^ The present findings suggest that PTGBD may represent a useful alternative for biliary drainage in patients with cholangitis complicated by DIC.

## 5. Limitations

This study has several limitations. First, because the number of cases was small and events were rare, adequate adjustment for confounders using multivariate analysis or propensity score methods could not be performed. In particular, significant differences existed in age and platelet count between groups, but statistical adjustment was difficult. In addition, several other baseline variables demonstrated SMDs >0.2, indicating meaningful imbalance between groups. These imbalances could not be addressed analytically due to the limited sample size and sparse-event structure. Second, as this was a single-center retrospective study, treatment introduction depended on the attending physician’s judgment, and selection bias may exist. Patients perceived as more severely ill – reflected by lower platelet counts and trends toward higher sequential organ failure assessment scores – were more likely to receive rTM. Consequently, treatment allocation was inherently confounded by clinical judgment, and residual confounding cannot be excluded even after descriptive comparisons. Large-scale prospective studies are required to confirm the efficacy and safety of rTM administration in biliary tract infections with DIC.

## 6. Conclusion

Perioperative rTM use during PTGBD was not associated with an increase in PTGBD-related AEs, including bleeding. These findings suggest that rTM may be administered without substantial additional procedural risk in patients with biliary tract infections and DIC, although confirmation in larger studies remains necessary.

## Author contributions

**Conceptualization:** Tetsushi Azami, Yuichi Takano.

**Data curation:** Tetsushi Azami, Naoki Tamai, Jun Noda.

**Formal analysis:** Tetsushi Azami.

**Funding acquisition:** Tetsushi Azami.

**Investigation:** Tetsushi Azami, Naoki Tamai, Jun Noda.

**Methodology:** Tetsushi Azami, Yuichi Takano, Fumitaka Niiya.

**Project administration:** Tetsushi Azami.

**Resources:** Tetsushi Azami.

**Software:** Tetsushi Azami.

**Supervision:** Tetsushi Azami.

**Validation:** Tetsushi Azami, Yuichi Takano.

**Visualization:** Tetsushi Azami.

**Writing – original draft:** Tetsushi Azami.

**Writing – review & editing:** Tetsushi Azami, Yuichi Takano, Naoki Tamai, Jun Noda, Fumitaka Niiya, Masatsugu Nagahama.
